# Analysis of Steel–Concrete Composite Beam with an Innovative Connector Made of Corrugated Metal Sheet and Shot Nails †

**DOI:** 10.3390/ma19091764

**Published:** 2026-04-26

**Authors:** Anna Derlatka, Paweł Kania

**Affiliations:** Faculty of Civil Engineering, Czestochowa University of Technology, Dabrowskiego 69 Str., 42-201 Czestochowa, Poland; pawel.kania@pcz.pl

**Keywords:** steel–concrete composite beam, connector, FEM, ADINA

## Abstract

This article analyses the possibility of the application of advanced fasteners in the construction of steel–concrete composite beams. The fasteners were made of corrugated metal sheet with a thickness of 1.00 mm, were in a dovetail shape, and had 2 or 4 shot nails in a single sheet fold. The nails were shot through the sheet into the flange of the steel I section. The 7.5 m-long beam was subjected to an experimental bending test. Based on the test results, the load-bearing capacity and failure mode of the beam were identified, enabling an evaluation of the feasibility of employing beams with innovative connectors in the construction of lightweight ceilings. A numerical model of the beam was constructed in ADINA using findings from experimental studies. A concrete material model, which considers both post-cracking and crushing behaviour and which enabled the identification of critical failure zones, was implemented. The beam connectors demonstrated sufficient load-bearing capacity, whereas the concrete slab was identified as the weakest component within the composite beam assembly. The proposed solution can be suitable for use as a fastener in steel–concrete composite ceiling structures in small utility public buildings. In accordance with Eurocode 4 requirements, the floor beam can carry a load of up to 5.7 kN/m^2^.

## 1. Introduction

When a new type of fastener is developed, testing is required. One way to do this is by using numerical calculations. Numerical simulations are widely used in steel–concrete composite structures under static [1,2,3] and dynamic loads [4]. In order to obtain reliable results, numerical models must adequately represent the components of composite structures [5,6,7]. Therefore, appropriate types of finite elements should be assumed. As the behaviour of composite beams exhibits significant non-linear effects, it is essential to properly model the interactions between the finite elements in such a way as to reflect the interactions between the components, especially the steel beam and slab, the steel beam and fasteners, and the slab and fasteners.

In [8], concrete slabs connected to steel beams by welded headed studs were tested. Calculations were performed using ANSYS software. The concrete slab, steel beam and shear connectors were simulated with C3D8R finite elements, but the reinforcing bars were simulated with truss elements. For accurate results, the same generations of mesh nodes were used for interactions between components, such as the bottom of the studs and the corresponding part of the steel beam, as well as the contact area between the studs and the concrete. The given contact conditions ensured sliding friction between the stud and the concrete surfaces. The coefficient of friction was 0.2. The same interaction was applied between the flange of the steel beam and the corresponding part of the concrete slab. The experience gained from the push-out tests of the connectors was utilised in both the experimental tests and numerical calculations of a full-size composite beam with head studs, as presented in [9].

The approach to simulating composite structures with bolts is similar to that for simulating an assembly with head studs. The numerical model presented in [10] was built using solid finite elements (C3D8R) to model the concrete slab, the steel beam, and the removable shear connectors. Truss-type elements (T3D2) were used to model the reinforcing bars. The surface-to-surface contact condition interactions available in the ABAQUS software were applied to all contact surfaces. The hard contact properties were used to simulate the behaviour of the contact in the normal direction to the contact plane, while, in the tangential direction, the penalty option was specified by assuming the coefficient of friction. At the interface between the components of the steel beam and the concrete slab, a coefficient of friction of 0.45 was assumed. For all other interactions, a coefficient of friction of 0.8 was used. Embedded constraints were used between the reinforcement and the concrete slab. As a result, the bars were embedded inside the slab by limiting the degrees of freedom of the truss element nodes. A very similar numerical model was built by the authors of [11], except that the coefficient of friction on the steel–steel contact surface was 0.2, and on the steel–concrete contact surface was 0.3.

A slightly different approach is presented in [12]. The numerical model of the composite beam was developed in ABAQUS. The slab was modelled using C3D8R solid elements. A quadrilateral shell element with reduced integration (S4R) was used to simulate the steel beam. The connectors were modelled using two-node beam elements (B31). The tendons and reinforcement were modelled using truss elements (T3D2). To represent the interaction, a hard property was assumed in the normal direction to the plane, and a penalty property was assumed in the tangential direction with a friction coefficient of 0.4.

It is worth paying attention to the approach presented in [13]. The steel–concrete bond force was modelled by using a nonlinear spring element. In order to simulate the bond-slip behaviour at the interface between steel and concrete, we utilised a model in the manner of the one presented in [14]. This is in contrast to [15], where, to enhance the accuracy of the FE model developed in Abaqus software version 6.14, the steel–concrete surface interaction was modelled as a cohesive behaviour.

Numerical simulations of composite structures made using composite dowel connectors developed in the ABAQUS program are included in [16,17,18]. On the other hand, ref. [1] presents the results of a numerical simulation of the composite beam using composite dowels made using the ADINA program. The reinforced concrete slab and the steel section were modelled using 3D solid elements. Reinforcing bars were modelled using truss elements. Mesh glueing was used between the connectors and the concrete slab, as well as between the connectors and the steel beam. The “mesh glueing” procedure involves joining two surfaces with different finite element meshes. Glueing leads to adhesion between glued surfaces and a smooth transition of movements. However, “rebar elements” were adopted to ensure the interaction between the reinforcement bars and the concrete slab. The developed numerical model was used to parameterise a composite beam 6.0 m long.

A numerical model of the composite beam, utilising hat connectors connected to the I beam via driven nails, was also created in the ADINA System program [19]. The steel profile, reinforced concrete slab, and nails were modelled using 3D solid elements, while the reinforcement bars were modelled using truss elements. The interaction between the reinforcement bars and the slab is ensured using “rebar elements”. The steel profile and the concrete slab were connected by “mesh glueing”. However, between the hat connectors made of sheet metal and the concrete slab, “rigid link”-type connections were used, which are constraints between two nodes: the master and slave nodes. When nodes move due to deformation, the slave node is constrained to move and rotate so that the distance between the master and slave nodes remains constant, and the rotations at the slave node are identical to those at the master node. Additionally, an advanced concrete material model, available in ADINA, was used to assess the possibility of concrete cracking. Thanks to this, the convergence of results was determined experimentally, numerically, and in accordance with the Eurocode 4 standard [20].

The described articles present the results of research on the numerical modelling of composite beams. Various software programs, such as ANSYS, ABAQUS, and ADINA System, were used for the simulations. Mainly, C3D8R elements were used to model the concrete slab and steel beam, and truss elements (T3D2) were used to model the reinforcement. Different approaches to interactions between components, such as surface contact with friction coefficients, were employed in various studies. The articles also discussed different methods of modelling connectors, including “mesh glueing” connectors, which allow a smooth transition of movements between surfaces with different finite element meshes. These works demonstrate various techniques and approaches for modelling and simulating the behaviour of composite structures, including their mechanical properties, enabling the optimisation of engineering designs in construction.

In steel–concrete composite structures, the corrugated trapezoidal sheet is a common tool for mechanically connecting the steel and concrete components. Most of them, in the top part of the fold of the sheet, have concave ribbing so that the concrete can be embedded in the sheet [21,22]. There are also solutions that use convex embossing on the top part of the fold, so the sheet can be embedded in concrete. As presented in [23,24], the friction connection is created thanks to the use of specially shaped sheet folds. The shape of such a cross-section improves the stability of the sheet and acts as hooks that connect to the concrete, preventing global buckling of the slab. An example of a corrugated sheet in the shape of a dovetail is shown in [25]. This sheet enables the construction of steel–concrete composite slabs without requiring additional span reinforcement in the form of bars. One of the advantages of the use of slabs on the corrugated sheet is that there is no need to make formwork, as the sheet performs this function. Sheet metal is relatively light, so moving it does not require heavy equipment. On the other hand, one of the disadvantages of dovetail-shaped sheets is its difficulty to transport, as the shape of the folds makes it impossible to stack one on top of the other.

**Figure 1:**
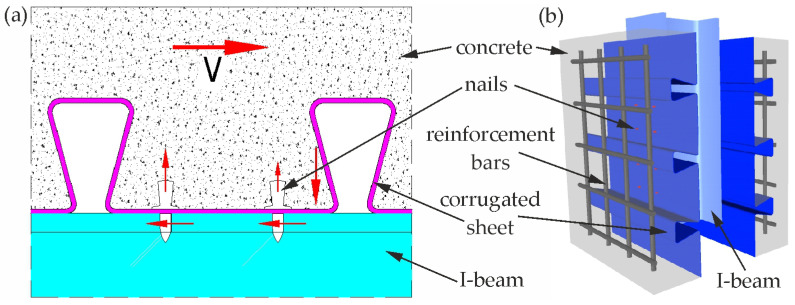
The connector for the composite beam [25]: (**a**) cross-section and (**b**) axonometric view of the sample for the push-out test.

In our previous paper [25], the load capacity of an innovative connector for steel–concrete composite structures made of corrugated sheet in the shape of a dovetail and driven nails was analysed (Figure 1). The nails were driven through the lower fold of the sheet into the flange of the steel I beam. The push-out test results demonstrated that the shape of the proposed sheet has a positive impact on the connection between the reinforced concrete slab and the steel I beam. At the same time, all fasteners analysed in [25], made of a metal sheet with a thickness of 1.00 and 1.25 mm and with two and four nails in the corrugation of the sheet, were considered ductile in accordance with the requirements of Eurocode 4 [20].

In our previous work, presented in a conference paper [26], we undertook the preliminary assessment of the possibility of applying innovative fasteners made from corrugated sheet in a dovetail shape and shot nails to create a steel–concrete composite beam. The 7.5 m-long beam was subjected to a bending test. The beam analysed in this paper was built using fasteners developed in [25], made from the 1.00 mm-thick corrugated sheet with two nails in the span zone and four nails in the support zone. The type (with two or four nails) and number of fasteners for the design composite beam were calculated in such a way that the total design resistance of the fasteners was equal to the design longitudinal shear force in the beam. The load capacity and failure mode of the beam were determined through experimental testing. Based on the results of tests conducted on the composite beam, a numerical model was developed in ADINA to accurately simulate beam behaviour. A concrete material model, taking into account the post-cracking and crushing behaviour, was used [27]. In this paper, the following results from the experimental tests were presented: the material test results and the beam bending test results. Furthermore, the following results from numerical simulation were shown: stresses and strains in the steel and concrete components, deflection of the composite beam and cracks in the concrete.

## 2. Experimental Program

Experimental tests were conducted on the raw materials of corrugated steel sheet, steel I beam, and concrete, as well as the steel–concrete composite beam made using innovative connectors.

### 2.1. Material Properties

**Figure 2:**
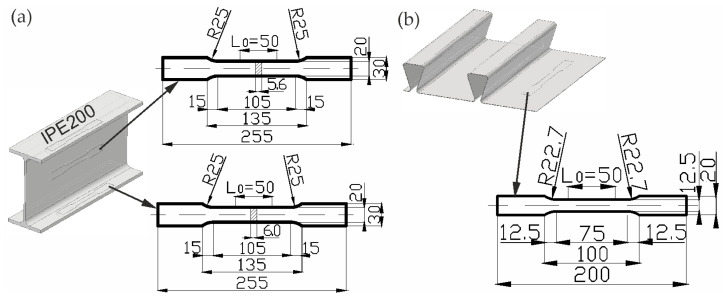
The samples for testing the material properties of (**a**) the I beam and (**b**) the corrugated sheet, mm.

The static tensile tests were performed for samples cut from the IPE 200 I beam made of S235JR steel grade marked in accordance with EN 10025-2 [28], and a corrugated sheet with a thickness of 1.00 mm made of S280GD steel, used to make the composite beam. The geometry of the samples (Figure 2) was developed on the basis of EN ISO 6892-1 [29].

C20/25 concrete class, marked in accordance with EN 206+A2 [30], was used to prepare the concrete slab. Tests of the compressive strength were performed. For the classification of concrete, the characteristic compressive strength was determined after 28 days of curing on cubic samples, with one side of 150 mm marked *f_ck.cube_*, in accordance with EN 206+A2 [30]. The tests were conducted on three samples taken from the concrete mixture.

### 2.2. Bending Test of Steel–Concrete Composite Beam

**Figure 3:**
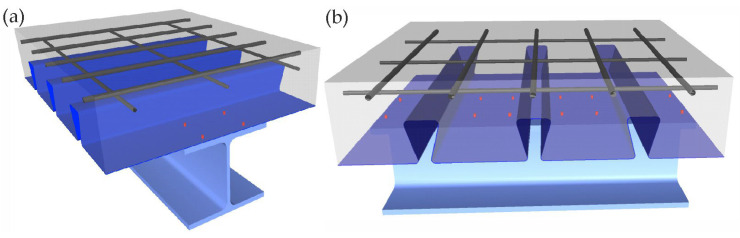
The schematic model steel–concrete composite beam: (**a**) view from the beam cross-section side and (**b**) view from the slab cross-section side.

**Figure 4:**
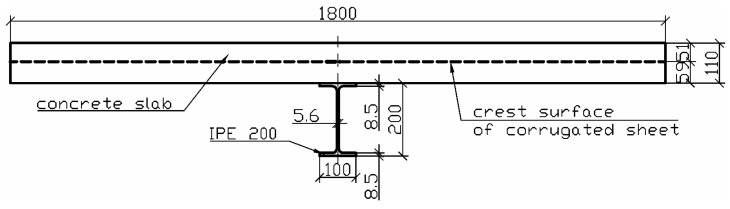
Cross-section of the analysed steel–concrete composite beam.

The next stage of experimental research was the verification of the previously developed connectors presented in [25] by making the steel–concrete composite beam. A schematic model of the beam in an axonometric view is shown in Figure 3. As shown in Figure 4, the beam was constructed from the IPE 200 I beam made of S235 structural steel. The corrugated sheets, galvanised on both sides, 1.00 mm thick, and made of S280GD steel, were connected to the I beam flange. It should be emphasised that the corrugated sheet has ribs on the top folds, which affect the adhesion of concrete to this part of the sheet. The sheet was fixed to the I beam with shot nails. The length of the beam was 7500 mm, while its width (corresponding to the width of the slab) was 1800 mm.

The experimental tests of the fasteners made of corrugated sheet and shot nails are presented in [25]. The summary of results for fasteners made of 1 mm sheet and two or four nails in one metal fold is shown in Table 1. The characteristic (*P_Rk_*) and design (*P_Rd_*) resistances of the fasteners were determined in accordance with EN 1994-1-1 [20]. Therefore, the design resistance *P_Rd_* was calculated from the following formula:

(1) $$P_{Rd}=\frac{f_{u}}{f_{ut}}\;\frac{P_{Rk}}{\gamma_{v}}$$

where

*f_u_*—the minimum specified ultimate strength of the connector material;
*f_ut_*—the actual ultimate strength of the connector material;
*γ_v_*—the partial safety factor for shear connection equal to 1.25.

**Table 1:** Summary of results from experimental tests of fasteners.

Type of Connector	4 Nails	2 Nails
Characteristic load capacity of the fastener *P_Rk_*, kN	40.50	27.00
Design resistance of the fastener *P_Rd_*, kN	32.40	21.60
Slip capacity *δ_uk_*, mm	7.70	10.77

The evaluation method outlined in EN 1994-1-1 [20] is primarily intended for the testing and assessment of typical headed studs manufactured from nominally homogeneous materials with well-defined mechanical properties. In such cases, the material parameters, including the minimum specified ultimate strength *f_u_*, can be directly related to standardised testing procedures and material specifications. However, the hybrid fasteners developed in this study differ fundamentally from typical headed studs. These consisted of two different materials: the sheet material and the driven nail material. These components exhibit different mechanical characteristics and their combined behaviour cannot be directly described using the provisions established for homogeneous connectors. In particular, there is no specified minimum ultimate strength *f_u_* for this type of hybrid, two-component connector within the current standard framework. Furthermore, due to the composite nature and geometry of the connector, it is not feasible to conduct a reliable and representative tensile test to accurately determine its ultimate strength. In view of these limitations, and in the absence of specific guidance in EN 1994-1-1 [20] for such connectors, a simplifying assumption was adopted. The ratio of the minimum specified ultimate strength of the connector material *f_u_* to its actual ultimate strength *f_ut_* was assumed to be 1.0 in order to calculate the design resistance *P_Rd_*.

Additionally, according to EN 1994-1-1 Eurocode 4 [20], shear connectors used in composite beams should exhibit sufficient ductility to ensure the effective redistribution of longitudinal shear forces along the steel–concrete interface. The connector may be classified as ductile if it demonstrates adequate deformation capacity prior to failure. This requirement is verified through push-out tests, in which the characteristic slip capacity is determined to be at least 6 mm. Both fasteners analysed in this paper were subjected to push-out tests, and the results are presented in [25]. As shown in Table 1, fasteners were considered ductile because the characteristic slip capacities *δ_uk_* were 7.70 and 10.77 mm, so they were of at least 6.0 mm [20]. Ductile connectors are characterised by a sufficient deformability to justify the perfectly plastic behaviour of the connection in the considered structure. Such a deformation capacity allows for stress redistribution among adjacent fasteners and structural elements.

Because the neutral axis of the steel–concrete composite beam was in the slab, the design longitudinal shear force in half of the beam span was equal to the design value of the normal force in the structural steel section, so 669.75 kN. The type and number of fasteners for the design composite beam were assumed in such a way that the total design resistance of the fasteners was like the design longitudinal shear force in the beam. The number of fasteners was also limited by the number of folds in the sheet. Finally, 10 fasteners with 4 nails in the support zones and 16 fasteners with 2 nails in half of the beam span were used, which confirms the following equation:

(2) 10×32.40 kN+16×21.60 kN=669.60 kN

**Figure 5:**
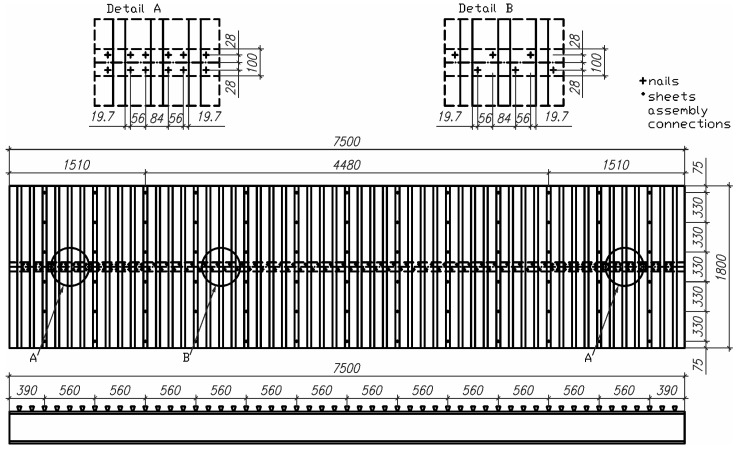
The detailed drawing of the composite beam.

The nails were placed in two rows. As shown in Figure 5, the row spacing was 56 mm. In the support zones (along the 1510 mm of the total length), 4 nails were used per sheet fold. On the other hand, in the span zone (along the 4480 mm of the total length), only 2 nails were used per sheet fold.

The corrugated sheet, in addition to the connector function, also provided permanent formwork for the monolithic reinforced concrete slab. The reinforced concrete slab was made of C20/25 class concrete. The thickness of the slab (above the folds of the sheet) was 51 mm, so that the total thickness of the plate was 110 mm. The slab width was 1800 mm, allowing it to cover the entire width of the profiled sheet.

The reinforcement mesh was prepared from ϕ8 mm bars made of steel with a characteristic yield strength *f_sk_* = 500 MPa and a ductility class according to PN-EN 1992-1-1 [31]. The transverse reinforcement was arranged according to the spacing of the sheet folds, i.e., every 140 mm. The longitudinal reinforcement bars are spaced at 125 mm intervals. The beam was concreted under fully supported conditions. The beam was matured in an air environment. Bending tests were conducted 28 days after the beam was fabricated.

**Figure 6:**
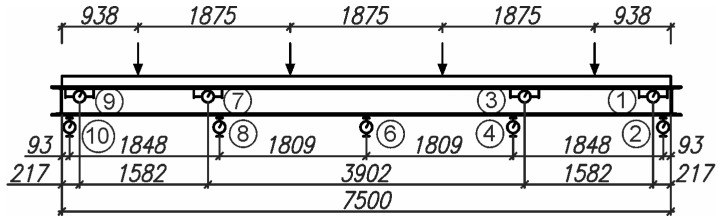
The scheme of arrangement of the load and dial gauges on the beam.

**Figure 7:**
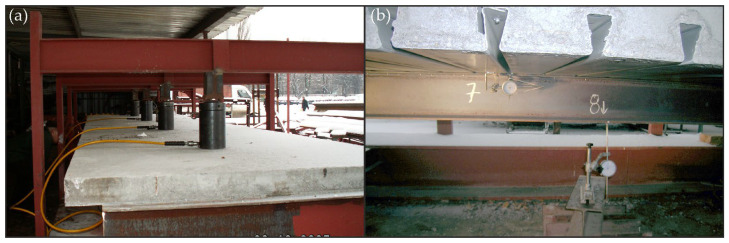
The beam bending test stand: (**a**) arrangement of cylinders and (**b**) dial gauges no. 7 and 8.

To ensure the static scheme of the analysed beam as a free-supported beam, the reinforced concrete slab was supported by two steel U200 profiles. The top surface of the slab was loaded with four pneumatic actuators in the form of cylinders, each with a 200 mm diameter. A pressure of 16.0 bar on each actuator, which corresponded to a force of 21.26 kN, was applied. On the lower surface of the corrugated sheet and the lower surface of the I beam, nine dial gauges were used to measure displacements. The spacing of the actuators and sensors is shown in Figure 6. Pictures of the test stand are shown in Figure 7.

A simply supported beam was used in the tests. As with other simply supported beams, maximum deflections were expected at mid-span. Therefore, it was decided to measure displacements using only a few sensors. Attention was paid to sensor no. 6, located at the mid-span of the beam, on the underside of the bottom flange of the I beam.

## 3. Numerical Model of Composite Beam

Based on the conducted experimental research, a numerical model was developed to reflect the behaviour of the analysed composite beam. Thanks to the numerical analysis, not only were the displacement-load diagrams obtained in experimental studies determined, but also detailed distributions of stresses and strains in individual components of the beam. In the numerical model of the floor beam, the finite element method was used to describe the influence of mechanical loads on deformations and stresses. Numerical calculations were performed using the ADINA program.

**Figure 8:**
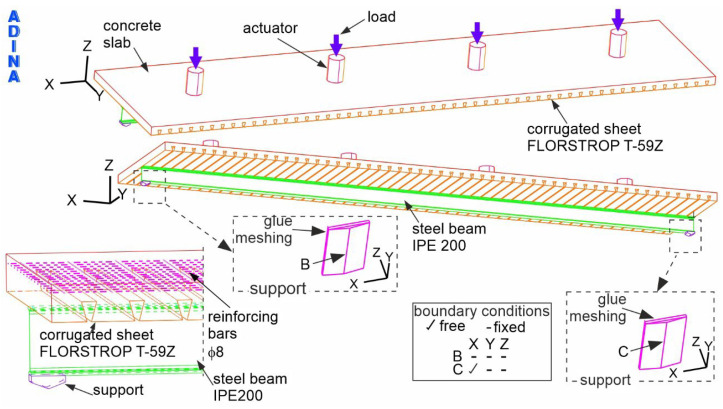
The numerical model of the composite beam.

A schematic diagram of the numerical model of the composite beam with marked boundary conditions is shown in Figure 8. The red, pink, orange, green and purple colours represent the concrete slab, reinforcement bars, corrugated plate, steel beam and supports, respectively. The boundary conditions reflect a simply supported composite beam. Along the bottom edge of one support, all degrees of freedom are blocked (symbol B). On the other hand, along the lower edge of the second support, the degrees of freedom along the X axis (symbol C) were released. The load was applied to four actuators, as indicated by the blue arrows. The spacing of the actuators was assumed to be the same as the spacing on the experimental test stand.

In the ADINA program, the type of analysis depends, among other factors, on the material models, contact conditions, and the type of applied load. In the numerical model of the composite steel–concrete beam, a non-linear analysis was used to account for large displacements and strains resulting from the adopted material models and the assumed load values.

**Figure 9:**
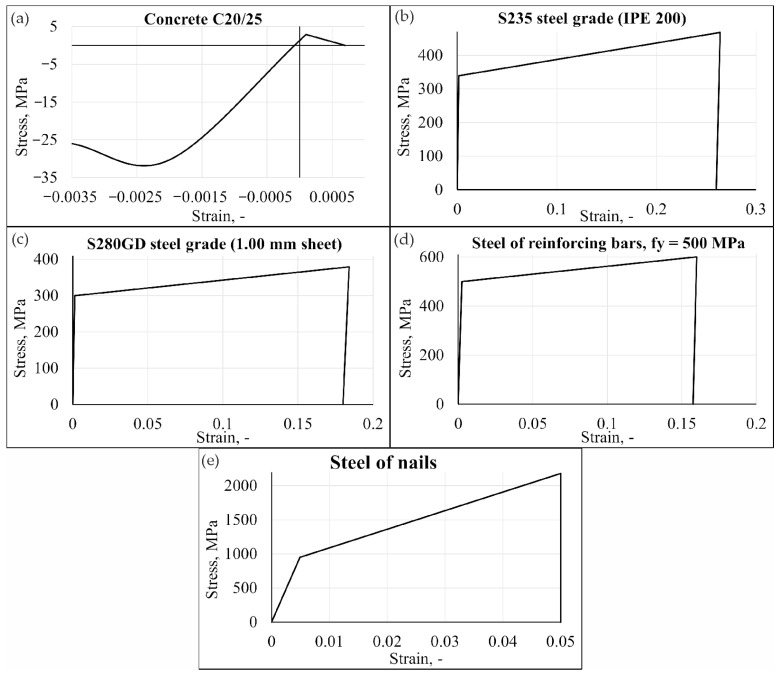
The material models of (**a**) concrete, (**b**) steel of IPE, (**c**) corrugated sheet, (**d**) steel of reinforcing bars, and (**e**) steel of shot nails.

The properties of the materials used in the numerical simulations are presented in Table 2 and Table 3. Material models were developed based on data obtained from experimental studies. Data not determined experimentally were assumed on the basis of the EN 1992-1-1 [31] and EN 1993-1-1 [32] standards. Steel components (I beam, sheets, nails, reinforcing bars) are described in the bilinear elastic-plastic model (von Mises yield criterion). The relationship between deformations and stresses is approximated by a curve that is defined by yield strength, modulus of elasticity and strain hardening modulus. The relationships are graphically shown in Figure 9b–e. An advanced concrete model available in the ADINA program was utilised to simulate concrete and assess the possibility of cracking. The uniaxial stress–strain relationship served as the basis for deriving the multiaxial stress–strain relationship. The material model curve of C20/25 class concrete is shown in Figure 9a.

The steel section and the concrete slab were modelled using eight-node 3D solid finite elements. Rebar elements were used to model the reinforcing bars. The sheets were modelled with eight-node shell elements, and the nails with two-node beam elements. Pneumatic actuators and supports were simulated using eight-node 3D-solid elements.

The interactions between the individual components in the finite element models were defined as follows. The connection between the sheet and the concrete slab was modelled using common nodes, ensuring full displacement compatibility at the interface.

The reinforcement bars were incorporated into the concrete slab using embedded rebar elements. ADINA can generate rebar elements combined with 3D solid elements (which were used to simulate the concrete slab). To achieve this, intersections of reinforcement lines and two-dimensional planes located on the sides of 3D solid elements are found. Then, at these intersections, nodes and rebar elements are generated, which connect subsequent nodes.

The connection between the I beam and the concrete slab, as well as between the nails and the slab, was also established by using common nail and I beam nodes.

Additionally, a “mesh glueing” technique was applied to model the interaction between the supports and the I beam, as well as between the actuators and the concrete slab, enabling the transfer of forces by non-matching meshes. The “mesh glueing” procedure, applied in ADINA, consists of joining two surfaces with different meshes of finite elements. “Mesh glueing” leads to adhesion between glued surfaces and a smooth transition of movements between them.

The adopted modelling assumptions can be considered adequate for capturing the overall structural response while maintaining relatively low computational demand. However, in the case of analyses aimed at a more detailed representation of local load transfer mechanisms within the proposed connector, a refinement of the contact formulation would be required. Such an enhancement would require more advanced contact definitions, thereby inevitably increasing computational demand.

## 4. Sensitivity Analysis of Numerical Model

**Figure 10:**
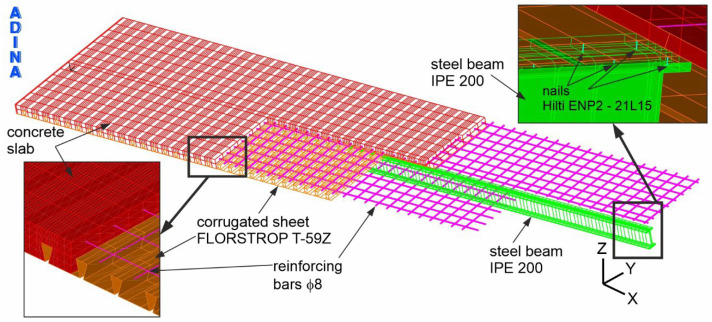
The mesh of the composite beam numerical model.

**Figure 11:**
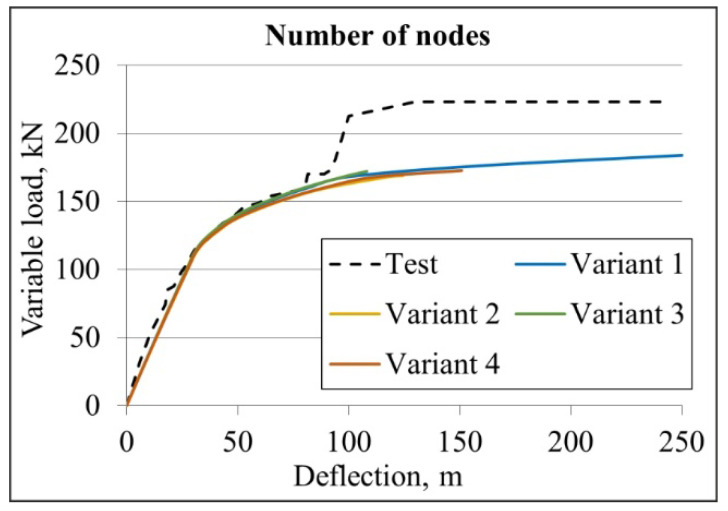
The deflection–external load graph for the assessment of numerical model sensitivity analysis due to the number of nodes.

The numerical model used to parameterise the steel–concrete composite beam was developed based on the results of the sensitivity assessment. The adopted numerical model mesh was shown in Figure 10. Four variants of numerical models of the beam were constructed, differing in the number of nodes in the finite elements of the steel beam, concrete slab, and corrugated sheet, as presented in Table 4. Table 4 presents the deflection values at two points corresponding to the external loads of 56 and 160 kN. Additionally, percentage references of deflections are presented. The deflection corresponding to 100% was assumed for model 1. The deflection–external load diagrams for each of the analysed variants are shown in Figure 11.

As shown in Figure 11, the numerical model of the composite beam with the small alloy is sensitive to the number of finite element nodes. The deflection values at the point corresponding to a load of 56 kN are similar, ranging from 100 to 101% when compared with the reference model. The deflection values at the point corresponding to a load of 160 kN ranged from 89.21 to 99.88 mm, which is 98–110% of the deflection of the reference model. The applied finite elements enabled convergence of the beam stiffness with the experimental results in the elastic range. At the same time, convergence between the simulation and experimental results was observed in the curvilinear range.

It was found that the eight-node shell elements, which simulate the corrugated metal sheet, provide sufficient accuracy while requiring less computational resources than the 16-node finite elements. It was decided to use 27-node 3D solid finite elements for the I beam and the concrete slab. These require more computing power than 20-node elements but allow for more accurate analysis of concrete elements. Therefore, variant 1 was selected for further analysis.

**Figure 12:**
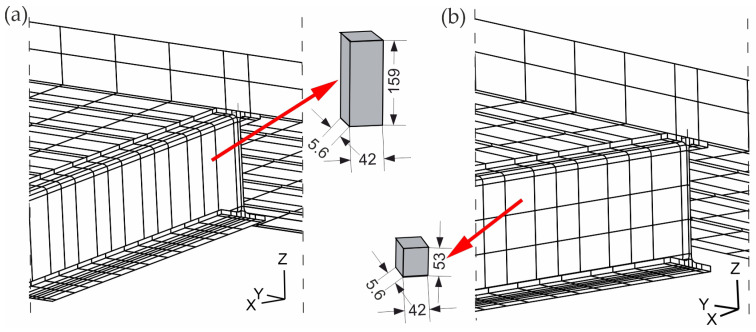
Mesh variants included in sensitivity analysis: (**a**) variant 1A and (**b**) variant 1B, mm.

The second criterion used to evaluate the sensitivity of the numerical model was the mesh size in the IPE beam web. The web was discretised using two different finite element meshes characterised by one or three elements per web height, as shown in Figure 12. The sensitivity analysis was conducted only for variant no. 1, corresponding to the first criterion of the sensitivity analysis, which is composed of 27-node 3D solid finite elements for the I section and concrete slab, and eight-node finite elements of the shell type for the corrugated sheet. In this case, variant nos. 1A and 1B were distinguished (Table 5).

**Figure 13:**
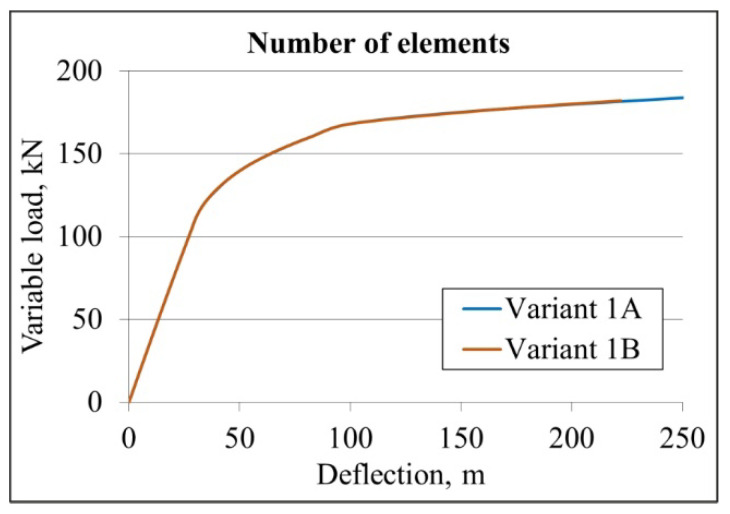
The deflection–variable load graph used for assessing sensitivity analysis due to the number of finite elements representing the steel web.

Deflection–external load diagrams for both variants are shown in Figure 13. The curves for variants 1A and 1B coincide, indicating that the numerical model of the composite beam is insensitive to the number of finite elements at the height of the steel beam web.

## 5. Results

As a result of the experimental tests, the mechanical properties of the materials used were obtained, and the bending load capacity of the composite beam was determined. In addition, the results from the numerical analysis of the beam are presented.

### 5.1. Material Properties

The following results were obtained from the static tensile test of the steel:

- the yield strength of each sample marked *R_e_* in accordance with EN ISO 6892-1 [29];
- nominal yield strength marked *f_y_* (according to EN 1994-1-1 [20]), which was taken as the mean value from experimental tests;
- tensile strength of each sample marked *R_m_* according to [29];
- nominal tensile strength marked *f_u_* according to [20], which was taken as the average value from experimental tests.

The test results of the IPE 200 I beam material used for the composite beam are presented in Table 6. IPE 200 section steel meets the requirements of the S235JR steel grade, as the average yield strength and tensile strength of 339 MPa and 468 MPa, respectively, meet the requirements of EN 10025-2 [28].

The material test results for the corrugated sheet used to make the composite beam are presented in Table 7. The average yield strength is 300 MPa, and it is higher than the required 280 MPa according to EN 10346 [33]. The average tensile strength is 379 MPa, and is higher than the 360 MPa required by the standard [33]. Therefore, the metal sheet material meets the requirements for steel grade S280GD.

Test results of the strength class of the concrete used for the construction of the composite beam are shown in Table 8. The average characteristic compressive strength *f_ck.cube_* for the tested concretes is 33.1, 32.2 and 30.3 MPa, respectively. In accordance with EN 206+A2 [30], all tested samples met the requirements for concrete class C20/25.

### 5.2. Experimental Test of Steel–Concrete Composite Beam

**Figure 14:**
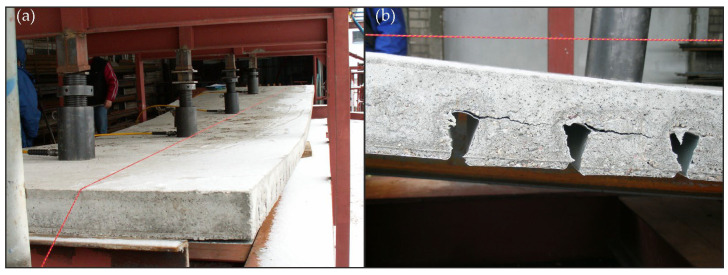
The composite beam (**a**) during the bending test and in (**b**) failure mode.

Pictures from the beam tests are shown in Figure 14. In the elastic range, no damage of the beam was observed, and in particular, no separation of the steel I beam from the slab was observed, which proves that the proposed solution of fasteners made of corrugated sheet and driven nails fulfilled its obligatory role. Moreover, in the elastic range, there was no delamination of the concrete slab from the corrugated sheet, confirming the effective composite action between the steel and concrete components. No visible concrete cracking was detected at this stage.

After exceeding the load of 117 kN, characteristic sounds of structural failure began to be heard, indicating the initiation of internal cracking and progressive damage within the concrete slab. At a load of 223.18 kN, a clear crack was observed in the concrete, shown in Figure 14b. The crack developed in the tensile zone at mid-span and propagated upward with increasing load, accompanied by a noticeable reduction in stiffness and an increase in deflection increments under subsequent loading steps. Ultimately, the composite beam was destroyed due to concrete tension cracking in the slab, leading to local detachments of the corrugated sheet from the concrete slab. These detachments were concentrated in the mid-span of the beam, in the region of maximum bending moment and stress concentration beneath the loading points.

**Figure 15:**
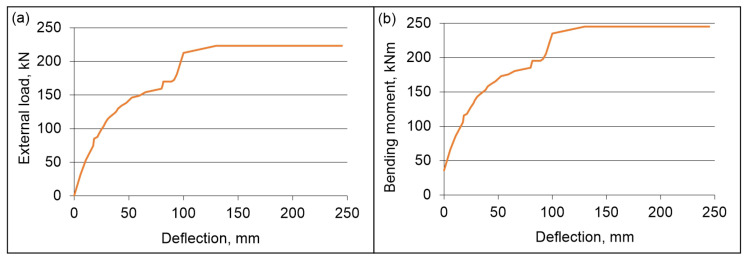
The beam test diagram: (**a**) displacement–external load and (**b**) displacement–bending moment.

The displacement–external load and displacement–bending moment diagrams are shown in Figure 15. It should be noted that the diagram presented in Figure 15a includes only the variable load and the corresponding displacement, i.e., coming only from the action of actuators. On the other hand, in Figure 15b, the bending moment due to the variable load, as well as the self-weight of the beam and cylinders, and the displacement as in Figure 15a were taken into account. The bending moment of the beam in the middle of its span, taking into account both the variable load and the self-weight of the cylinders (the mass of a single cylinder was 36 kg, which corresponds to 0.353 kN), as well as the beam’s own weight, which is equal to 4.922 kN/m, was calculated according to the following formula:

(3) $$M=\frac{\left(P+0.353\;kN\right)\cdot 7.5\;m}{2}+\frac{4.922\frac{kN}{m}\cdot {\left(7.5\;m\right)}^{2}}{8}$$

where *P*—variable load.

It was observed that the beam carries the load in the elastic range up to a force of 116.9 kN, which corresponds to a bending moment of 145.53 kNm and a deflection of 32.49 mm. At the same time, this corresponds to a surface load of 8.6 kN/m^2^. Once these values are exceeded, the graph becomes curved, and the beam enters a plastic state. At a load of 170 kN, a clear flow was observed. The load value remained constant and during this time the displacement increased by 7.36 mm. A similar phenomenon was observed at a load of 223.18 kN, with a deflection increase of 115 mm.

### 5.3. Numerical Model of Composite Beam

**Figure 16:**
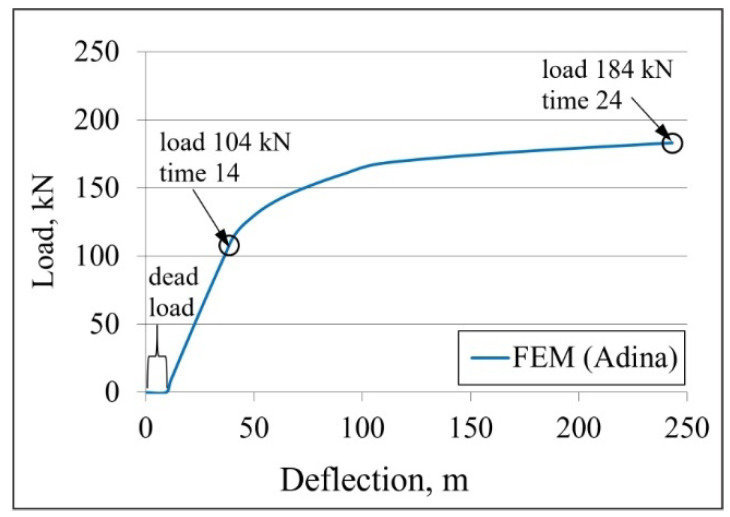
The deflection–load diagram from the numerical simulation of the composite beam.

The deflection–force diagram obtained from a numerical simulation of the composite beam with innovative fasteners, in the form of the corrugated sheet and shot nails, is shown in Figure 16. The deflection at the midspan of the beam, due to the variable load (four actuators) and the dead weight, was considered. The vertical axis shows only the external load (four actuators).

The curve begins with a rectilinear section, characterised by a variable load of 0 kN and a displacement range of 0–9.26 mm. This is an effect derived from the self-weight of the beam and actuators. Then the curve assumes a rectilinear character with a simultaneous increase in displacement and load. After the beam transitions from the elastic state to the plastic state, i.e., after exceeding a load of 104 kN, the graph curves and the load increase non-linearly with respect to the displacement. The beam calculations ended at the point corresponding to the variable load of 184 kN.

**Figure 17:**
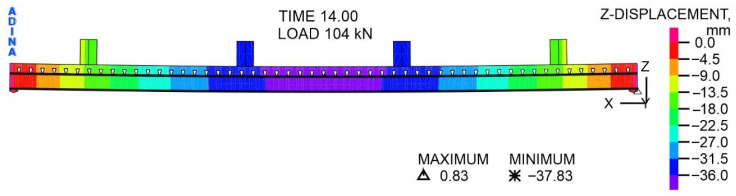
The distribution of displacements with respect to the *Z*-axis, at a load of 104 kN, mm.

The distribution of displacements of the steel–concrete composite beam relative to the *Z*-axis at a variable load of 104 kN is shown in Figure 17. The presented displacements result from the combined influence of the total load, i.e., the variable pressure from the actuators, and the permanent load from the self-weight of the beam and actuators. The displacements in the vast majority of the beam take negative values, i.e., the beam moves in the direction of gravity. On the other hand, in the support zones, the displacements take on positive values, i.e., the beam is moved upwards by a maximum of 0.83 mm. The maximum deflection occurs in the middle of the span, at the edge of the concrete slab, and is 37.83 mm.

**Figure 18:**
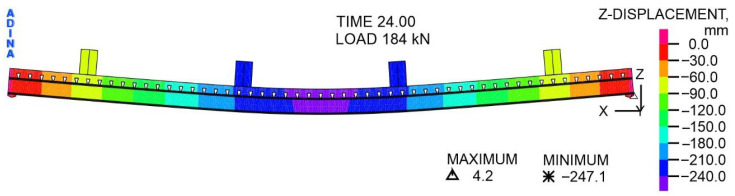
The distribution of displacements with respect to the *Z*-axis, at a load of 184 kN, mm.

The distribution of vertical displacements of the composite beam along the *Z*-axis at a variable load of 184 kN is shown in Figure 18. The distribution of displacements is similar to that shown in Figure 17. However, in the support zones, the displacements take positive values, with a maximum of 4.2 mm. The maximum deflection occurs at the midpoint of the span, at the edge of the concrete slab, and is equal to 247.1 mm.

**Figure 19:**
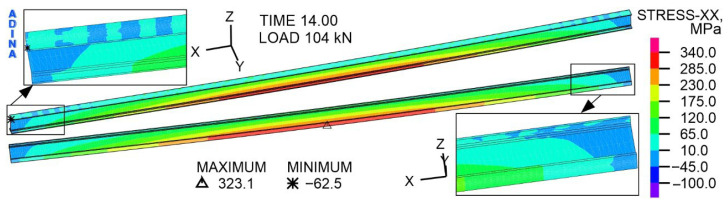
The distribution of stress with respect to the *X*-axis in the steel component of IPE 200, at a load of 104 kN, MPa.

**Figure 20:**
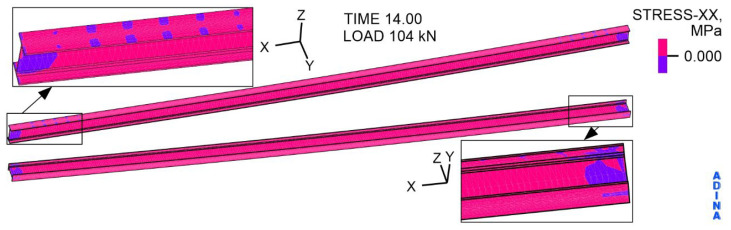
The distribution of stress with respect to the *X*-axis in the steel component of IPE 200, at a load of 104 kN, MPa.

The distribution of directional stresses with respect to the *X*-axis in the IPE I beam is presented in Figure 19 and Figure 20. Under a load of 104 kN, the middle part of the IPE is in tension. In the support zones of the I beam, the web is in compression, and both flanges are in tension. At the same time, in the support area, the top flange, where the nails enter, is compressed. The extreme of the I beam tensile stress occurs in the middle of the bottom flange span and is 323.1 MPa. This value is slightly lower than the determined yield strength of the I beam material, which is 339 MPa (Table 6). The compressive stress extreme of 62.5 MPa is in the top flange, in the support zone.

**Figure 21:**
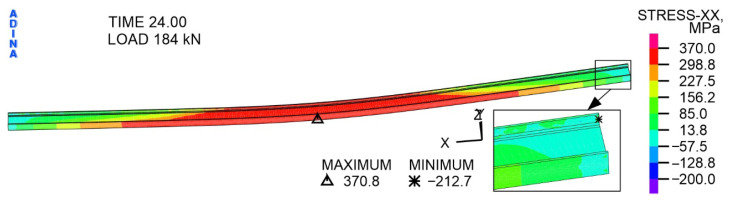
The distribution of stress with respect to the *X*-axis in the steel component of IPE 200, at a load of 184 kN, MPa.

**Figure 22:**
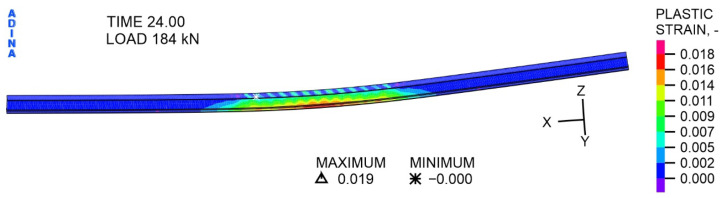
The distribution of plastic strain in the steel component of IPE 200, at a load of 184 kN, -.

The character of the distribution of directional stresses with respect to the *X*-axis in the IPE 200 steel I beam at a variable load of 184 kN (Figure 21) is similar to the distribution at a load of 104 kN. It is worth noting that the stress values increased only slightly, with the tensile stress extreme at 370.8 MPa and the compressive stress extreme at 212.7 MPa. The most important thing is that almost the entire central part of the beam is covered in red, i.e., values of tensile stresses exceeding the determined yield point of 339 MPa. At the same time, a plastic hinge is formed in the middle part of the steel beam, as evidenced by the plastic deformations shown in Figure 22.

**Figure 23:**
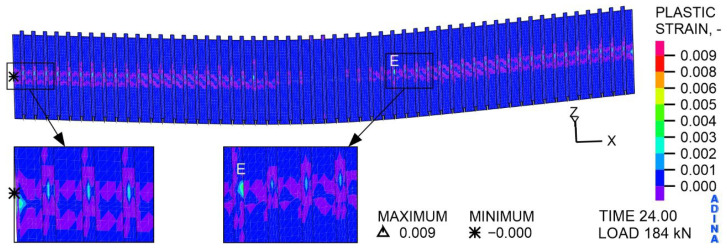
The distribution of plastic strains in the sheet, at a load of 184 kN, MPa.

As shown in Figure 23, deformations of the corrugated sheet were also created at a variable load of 184 kN. These occur in the middle of the sheet width, i.e., in the area of contact with the steel beam. A clear concentration of plastic deformations in the sheet can be observed in the support zone and at point E, specifically on the inside of the sheet fold, corresponding to the actuator’s action on the concrete slab.

**Figure 24:**
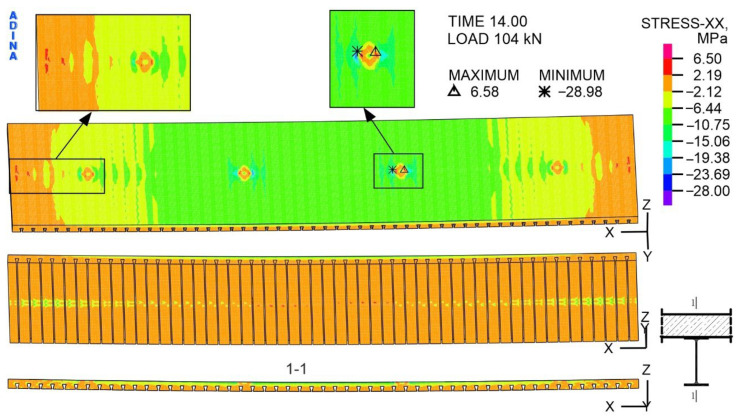
The distribution of stress with respect to the *X*-axis in the concrete slab, at a load of 104 kN, MPa.

As shown in Figure 24, the compressive stresses in the span zone occurred on the top surface of the slab. The exceptions are the pressure points of the actuators, where local concentrations of tensile stresses were observed. The top surface of the concrete slab in the support zone is stretched. On the bottom surface of the slab, two colours can be distinguished: yellow, corresponding to compressive stresses and which is located in the middle of the width of the slab, and orange (tensile stresses), which occurs in the remaining area. The maximum compressive stress in the slab is located on the top surface and amounts to 28.98 MPa. This value does not exceed the determined concrete compressive strength of 30.3 MPa (Table 8). The maximum tensile stress in the slab is 6.58 MPa, which is higher than the assumed tensile strength of 2.2 MPa.

**Figure 25:**
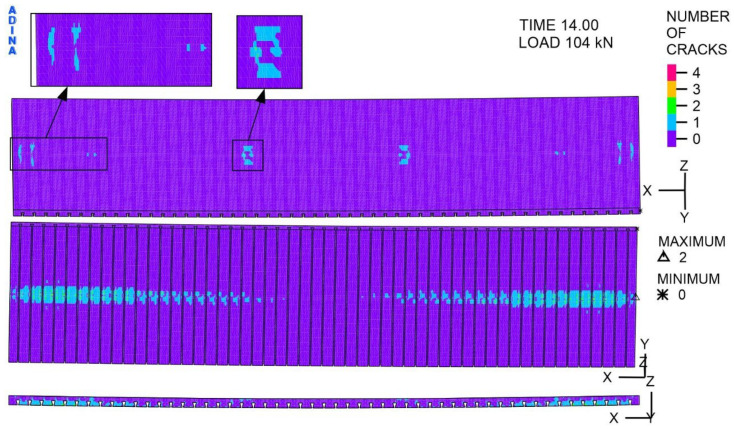
The cracks in concrete, at a load of 104 kN, -.

The concrete material model available in the ADINA program enables the verification of crack locations in concrete finite elements. The parameter defined as “number of cracks” allows four values, where 1 indicates the presence of open cracks, 2 denotes the occurrence of closed cracks, 3 represents concrete crushing, and 4 indicates the presence of cracks. In the slab, cracks appeared at places where the limit tensile stresses were exceeded (Figure 25). These are located mainly on the bottom surface of the slab, in the middle part of the width, along the entire span. There are open and closed cracks. In addition, on the top surface of the slab, in the support zones, and at the actuator pressure locations, open cracks marked with the number 1 were observed.

**Figure 26:**
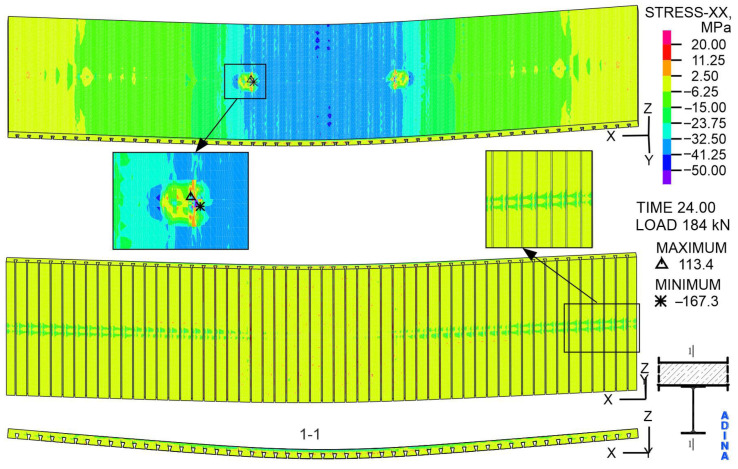
The distribution of stress with respect to the *X*-axis in the concrete slab, at a load of 184 kN, MPa.

The character of the directional stress’s X distribution on the top surface of the slab under a load of 184 kN (Figure 26) is also similar to the distribution occurring at a load of 104 kN. However, the stress values in the inter-span zone are higher because the compression extreme is equal to 167.3 MPa. The tensile extreme also occurs on the top surface of the slab and is equal to 113.4 MPa. This value exceeds the concrete compressive strength of 30.3 MPa. On the other hand, the bottom surface of the slab is mostly stretched, which is marked in yellow, and the stresses do not exceed 2.5 MPa. At the midpoint of the width, where it contacts the steel beam, the slab is compressed, with compressive stress values reaching up to 24 MPa.

**Figure 27:**
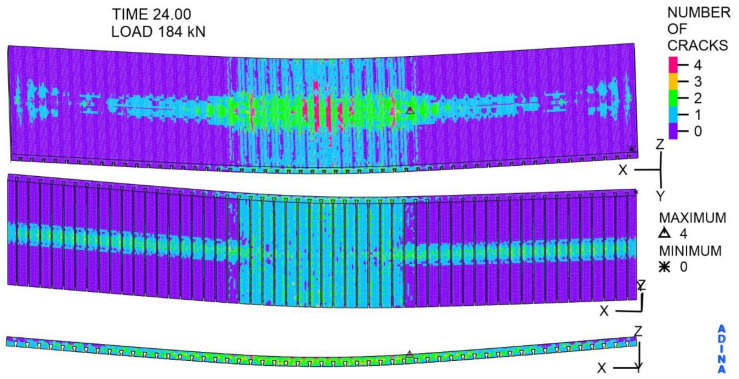
The cracks in the concrete, at a load of 184 kN, -.

Significantly exceeding the tensile strength of the concrete, which is 2.2 MPa, results in numerous cracks in the concrete, shown in Figure 27. The most significant fact is that cracks marked with the number 4 are on the top surface of the slab. Two characteristic areas can be distinguished on the lower surface of the slab: the centre of the width, along the entire span, and the centre of the span, along its entire width. In these areas, there are cracks marked with numbers 1, 2, and 3, corresponding to the occurrence of open and closed cracks, and concrete crushing.

## 6. Discussion

**Figure 28:**
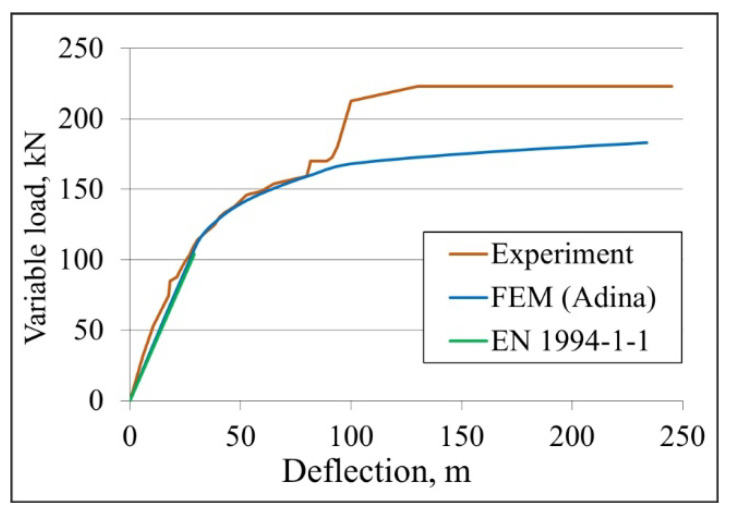
The deflection–load graph comparing results obtained experimentally, numerically and calculated according to the standard EN 1994-1-1 [20].

A comparison of the deflection–load diagrams for the composite beam obtained experimentally (orange) and numerically (blue) is shown in Figure 28. Additionally, the curve determined in accordance with the EN 1994-1-1 [20] standard is marked in green. It is easy to notice the convergence of the curves in the initial phase of the graph, i.e., in the elastic range. This demonstrates that the experimentally determined stiffness of the composite beam is similar to that of its numerical equivalent. However, the load at which the beam changes from elastic to plastic, as determined based on the results from experimental tests, is 116.9 kN. However, the force determined by numerical simulation is approximately 10% lower, as it is equal to 104 kN. The difference is due to the tolerances of the beam manufacturing, which were 1 mm for steel parts and 10 mm for concrete parts. Simultaneously, the developed numerical model had nominally perfect geometry, with neither geometric nonlinearity nor the tolerance of beam manufacturing.

In most numerical models, a nominally perfect geometry is assumed primarily to isolate the fundamental structural behaviour. By removing geometric imperfections and nonlinear effects, the internal mechanical response of the structure can be studied without additional factors. Starting from a perfect configuration makes it easier to understand the essential mechanics before introducing secondary effects. Another important reason is that real geometric imperfections are inherently uncertain. Manufacturing tolerances, residual stresses, and thickness variations vary from one member to another and are usually random. Assuming nominally perfect geometry improves numerical robustness and efficiency. Therefore, a perfect geometry serves as a standardised reference configuration.

The maximum load transferred by the beam during the experiment is 223 kN, whereas the numerically determined value is 184 kN, representing a 21% reduction. The adopted material models, especially the concrete model, contribute to the difference. The calculations ended after the cracks occurred, marked as number 4, i.e., after the concrete on the top surface of the slab cracked in the area of the actuators’ pressure. It should be noted that, even under the action of a load of 104 kN in the concrete slab, the tensile stresses at the contact point of the slab with the actuators are 6.58 MPa and exceed the ultimate tensile strength of the concrete, which is equal to 2.2 MPa. This is only a local stress concentration caused by a concentrated load. With a load of 104 kN in the remaining area of the slab, the compressive and tensile stresses do not exceed the concrete ultimate strength, indicating that the structural response remains within the safe range. The elevated stresses are confined to a small region directly under the load application point and decrease rapidly with increasing distance from this location. In the final step of the calculations, representing a load of 184 kN, a similar dependence of the local concentration of compressive and tensile stresses at the point of action of the concentrated load was observed. However, the values of these stresses were much larger.

The numerical results indicate that failure initiation is governed not by global bending or shear mechanisms, but by localised overstressing caused by the concentrated load transfer. To mitigate the crushing effect of concrete at the pressure point of the actuators, subsequent research suggests using a flat plate between the actuators and the slab. Such a plate would redistribute the applied forces over a larger area, effectively transforming the concentrated loads into a surface load. This change would greatly lower peak stresses in the contact area, encourage a more even stress distribution, and better reflect the load transfer conditions typical in real-world scenarios.

The results of numerical calculations demonstrate that the proposed solution for the composite steel–concrete beam with connectors, utilising a corrugated sheet and nails, satisfies the following basic assumptions: the steel I beam is predominantly in tension, while the concrete slab is in compression. The local concentration of compressive stresses in the steel beam and tensile stresses in the concrete slab occurs in the support zone, a consequence of the assumed boundary conditions.

The maximum tensile stress in the slab under a load of 104 kN is 6.58 MPa, which is higher than the assumed ultimate tensile strength of 2.2 MPa. Therefore, cracks appear in the slab and are located on its bottom surface. There are open and closed cracks in this area. Additionally, open cracks were observed on the top surface of the slab in areas where the actuators were applied. The reason is the adopted boundary conditions and the absence of a flat plate between the actuators and the slab, which could reduce the direct pressure effects of the actuators on the concrete slab.

The analysed composite beam behaves elastically up to a load of 116.9 kN, which corresponds to a design floor load of 8.6 kN/m^2^. Assuming a partial factor of 1.5 according to Eurocode 4 [20], a floor beam can carry a characteristic load of 5.7 kN/m^2^. Therefore, the proposed fastener solution for composite structures met the expected requirements. The connectors in the beam demonstrated sufficient load capacity, and the weakest component of the composite beam proved to be the slab. This form of the sheet cross-section allows the transfer of delamination forces between the steel beam and the concrete slab, without the need for additional headed connectors.

Additionally, the corrugated sheet in the dovetail shape enables the construction of steel–concrete composite slabs without the need for additional span reinforcement in the form of traditional reinforcing bars. The geometry of the ribs ensures effective mechanical interlock between the steel sheet and the concrete, allowing the sheet to act simultaneously as permanent formwork and as tensile reinforcement after curing. As a result, the construction process is significantly simplified. The execution of slabs poured on a steel sheet is faster than that on traditional formwork. This reduction in labour intensity translates directly into shorter construction time and lower overall project costs. At the same time, when compared with a conventional flat reinforced concrete slab of similar span capacity, slabs cast on corrugated sheets are generally lighter. The profiled geometry reduces the volume of concrete in the tension zone. The decreased self-weight not only reduces the load transferred to supporting elements such as beams but can also lead to savings in columns or foundation design and overall structural dimensions.

On the other hand, certain disadvantages must also be considered. One of the primary drawbacks concerns transportation and storage. Due to the shape of the dovetail folds, the sheets cannot be stacked compactly one on top of another, which increases the required storage space and complicates logistics. This may result in higher transportation costs and more demanding on-site handling. In addition, the profiled geometry can make cutting and detailing at supports more complex compared to flat sheets.

Unlike classic headed stud fasteners, which require a special device powered by high current to make fasteners in the form of a corrugated sheet and driven nails, this process can be achieved without such a device. Traditional stud welding requires access to an appropriate power supply, trained personnel, and strict safety procedures, which may limit its applicability to smaller projects or sites with limited technical facilities. In contrast, devices for driving nails are commonly available both in construction factories and directly on building sites. They are widely used in various types of building structures. An additional advantage is that many nail-driving devices operate without the need for an external electrical power source, relying instead on mechanical or pneumatic systems. This increases their mobility and makes them particularly suitable for on-site conditions. The simplicity and speed of installation can significantly reduce assembly time compared to welded stud connectors.

Accordingly, fasteners made of corrugated steel sheet and driven nails can be used as fasteners for steel–concrete composite structures, particularly when applied in lightweight floor systems for small public utility buildings. In such applications, the structural demands are often moderate, and the benefits of reduced installation complexity, lower cost, and improved accessibility can outweigh the higher load capacity typically associated with welded studs.

Steel–concrete composite beams are mostly designed as single-span, simply supported members. The reason this assumption is related to the distribution of bending moments. In simply supported beams, only positive bending moments occur, so the concrete slab remains predominantly in compression. This is advantageous because concrete performs well under compressive stresses. However, in continuous beams used in reinforced concrete structures, negative bending moments develop over the supports, placing the concrete slab in tension. As concrete has low tensile strength, this leads to cracking and reduces the effectiveness of the concrete action in these regions. As a result, additional reinforcement and more sophisticated design approaches are required. From a design perspective, simply supported composite beams are preferred because they allow straightforward analysis and the direct application of standard procedures, such as those provided in EN 1994-1-1 Eurocode 4. The internal force distribution is easier to determine, and the assumptions related to shear connection and plastic redistribution are more readily satisfied. This contributes to both reliability and efficiency in the design process.

The developed and validated parametric numerical model of the steel–concrete composite beam can be used to generate a database for further analysis employing artificial intelligence–based methods such as artificial neural networks (ANNs) and genetic algorithms (GAs). By systematically varying selected input variables, the model enables the efficient generation of large datasets describing ultimate capacity and stiffness. These datasets can then serve as training and validation sets for ANN models, enabling the development of fast predictive tools that approximate complex nonlinear structural behaviour with high accuracy. At the same time, genetic algorithms can be applied to perform optimisation tasks, such as identifying optimal design configurations that maximise load-bearing capacity while minimising material consumption or cost. The numerical model becomes not only a tool for deterministic simulation but also a foundation for advanced data-driven analysis and structural optimisation.

## 7. Conclusions

1. The analysed composite beam behaves elastically up to a load of 116.9 kN, which corresponds to a design floor load of 8.6 kN/m^2^. According to the requirements in accordance with Eurocode 4, a floor beam can carry a characteristic load of 5.7 kN/m^2^.
2. Based on the results of experimental tests, it was found that the beam connectors showed sufficient load-bearing capacity, and the concrete slab was the weakest component of the composite beam. The beam was damaged due to cracking in the slab, resulting in local detachments of the corrugated sheet from the concrete slab.
3. The numerical calculations carried out showed that the mechanism of concrete slab failure is related to the formation of a plastic hinge in the middle of the span of the steel beam and plastic deformations occurring in the corrugated sheet in the middle of its width. Plastic deformations of the top and lower folds of the sheet contributed to delamination between the sheet and the concrete slab.
4. Based on the experimental tests and numerical calculations, it was found that the proposed solution of fasteners made of corrugated sheet and shot nails can be used as the fastener for steel–concrete composite structures applied in the lightweight ceilings of small utility public buildings.
5. The developed numerical model of the composite beam allows the optimisation of the beam cross-section and the optimisation of the connector depending on the assumed boundary conditions, the manner and size of the acting load.

## Figures and Tables

**Table 2:** The properties of the steel used in the numerical model of the beam.

Property	Value			
	Steel S235 (IPE 200)	Steel S280GD (1.00 mm Thick)	Reinforcement Steel f_sk_ = 500 MPa	Steel Nails
Young’s modulus *E*, GPa	210	210	205	193
Yield strength *f_y_*, MPa	339	300	500	950
Ultimate tensile strength *f_u_*, MPa	468	379	600	2180
Poisson’s ratio *ν*	0.3	0.3	0.3	0.3
Density, kg/m^3^	7850	7850	7850	8000
Minimum elongation *A*_5_, %	26	18	16	5

**Table 3:** The properties of the concrete used in the numerical model of the beam.

Property	Value of Concrete C20/25
Tangent modulus at zero strain *E_cm_*, GPa	30
Uniaxial cut-off tensile stress *f_ctm_*, MPa	2.2
Uniaxial maximum compressive stress *f_cm_*, MPa	−30.3
Uniaxial compressive strain *ε_c_*_1_, ‰	−2
Uniaxial ultimate compressive stress *f_ck_*, MPa	−23
Uniaxial ultimate compressive strain *ε_cu_*_2_, ‰	−3.5
Density *ρ*, kg/m^3^	2500
Poisson’s ratio *ν*	0.2

**Table 4:** Sensitivity analysis due to the number of nodes in finite elements.

Variant		1	2	3	4
Number of nodes in element group	Steel beam (3D solid elements)	27	27	20	20
	Concrete slab (3D solid elements)	27	27	20	20
	Steel sheet (shell elements)	8	16	8	16
	Nails (beam elements)	2	2	2	2
	Actuators and supports (3D solid elements)	8	8	8	8
Deflection under load 56 kN, mm		24.15	24.46	24.03	24.27
Deflection under load 56 kN, %		100%	101%	100%	101%
Deflection under load 160 kN, mm		91.09	99.88	89.21	97.76
Deflection under load 160 kN, %		100%	110%	98%	107%

**Table 5:** Number of finite elements used for sensitivity analysis.

Element Group	Number of Finite Elements	
	Variant 1A	Variant 1B
No 1 (3D solid) representing a steel beam	2754	8262
No 2 (3D solid) representing a concrete slab	6450	6450
No 3 (rebar) representing reinforcing bars	4824	4824
No 4 (beam) representing nails	912	912
No 5 (shell) representing sheet metal	5610	5610
No 6 (3D solid) representing actuators	16	16
No 7 (3D solid) representing supports	4	4
Total	20,570	26,078

**Table 6:** The results from static tensile tests of IPE 200 steel.

Sample No	Yield Strength *R_e_*, MPa	Tensile Strength *R_m_*, MPa
IPE 200.S1 (web)	349	468
IPE 200.S2 (web)	344	468
IPE200.P1 (flange)	333	-
IPE200.P2 (flange)	331	-
$\mathrm{Average}\;\bar{x}$:	*f_y_* = 339	*f_u_* = 468

**Table 7:** The results from the test of corrugated sheets.

Sample No	Yield Strength *R_e_*, MPa	Tensile Strength *R_m_*, MPa
T-59.1	303	384
T-59.2	301	378
T-59.3	297	374
$\mathrm{Average}\;\bar{x}$:	*f_y_* = 300	*f_u_* = 379

**Table 8:** Test results of the concrete class used to make the composite beam.

Sample No	Characteristic Compressive Strength *f_ck.cube_*, MPa
5678K1	31.8
5678K2	29.0
5678K3	30.2
$\mathrm{Average}\;\bar{x}$:	*f_ck.cube_* = 30.3

## Data Availability

The original contributions presented in this study are included in the article. Further inquiries can be directed to the corresponding author.
